# Association of Peripheral Blood Parameters With Outcomes of COVID-19 Infection in a Tertiary Care Setting of Eastern India: An Institute-Based Study

**DOI:** 10.7759/cureus.20745

**Published:** 2021-12-27

**Authors:** Jitendra S Nigam, Anup Kumar, Ruchi Sinha, Haripriya H, Neeraj Kumar, Surabhi ., Tarun Kumar, Shreekant Bharti, Punam P Bhadani

**Affiliations:** 1 Pathology and Lab Medicine, All India Institute of Medical Sciences, Bibinagar, Telangana, IND; 2 Orthopedics, All India Institute of Medical Sciences, Patna, IND; 3 Pathology and Lab Medicine, All India Institute of Medical Sciences, Patna, IND; 4 Community and Family Medicine, All India Institute of Medical Sciences, Patna, IND; 5 Trauma and Emergency, All India Institute of Medical Sciences, Patna, IND

**Keywords:** blood, covid-19, neutrophils, lymphocytes, coronavirus

## Abstract

Background

Severe acute respiratory syndrome coronavirus 2 (SARS-CoV-2) caused the Coronavirus DISEASE 2019 (COVID-19) pandemic. Blood investigations play a vital role in providing information regarding the inflammatory process. Previous studies have shown that complete blood count parameters have clinical importance in predicting disease outcomes. However, there is a scarcity of literature published from our region in India.

Aims

The present study was conducted to describe the epidemiological, clinical, and hematological characteristics and outcomes of COVID-19 confirmed cases.

Material and methods

All real-time reverse transcriptase-polymerase chain reaction (RT-PCR) confirmed SARS-CoV-2 cases admitted in our institute over three months, from July to September 2020, were included in the study population. The blood samples of SARS-CoV-2 positive cases were analyzed for complete blood counts and coagulation profile on admission and at the time of discharge (most recent in case of mortality).

Results

A total of 252 RT-PCR confirmed SARS-CoV-2 cases were included in the study. The most common age group affected was 46 to 60 years, and the male-to-female ratio was 2.45:1. The most common clinical symptom was dyspnea, and the commonest comorbidity was hypertension. The statistical analysis showed a significant association of age, absolute neutrophil count (ANC) D-dimer, neutrophil-to-lymphocyte ratio (NLR), and platelets-to-lymphocyte ratio (PLR) with intensive care unit (ICU) admission and death. Gender, dyspnea, and absolute eosinophil count (AEC) showed significant association with ICU patients only, while liver disease and absolute lymphocyte count (ALC) had a significant association with death.

Conclusion

There are many notable clinical and hematological manifestations of COVID-19. Age, gender, dyspnea, comorbid liver disease, ANC, ALC AEC, NLR, PLR, and D- dimer may help clinicians predict the disease progression and reduce mortality risk.

## Introduction

Severe acute respiratory syndrome coronavirus 2 (SARS-CoV-2) is the name given by the International Committee on Taxonomy of viruses to the betacoronavirus, which is the causative agent of the coronavirus disease 2019 (COVID-19) [1-3]. This highly contagious RNA virus of the coronaviridae family is transmitted mainly via droplets and contaminated surfaces [1-3]. The World Health Organization (WHO) declared the infection caused by SARS-CoV-2 as a pandemic in March 2020 [1,2]. All coronavirus strains cause acute respiratory distress syndrome (ARDS) and fatal acute lung injury [2]. SARS-CoV-2 binds to its receptor angiotensin-converting enzyme 2 (ACE2) present on ciliated cells and type 2 pneumocytes of airways, the endothelium of blood vessels, and other body tissue [2,3]. The clinical spectrum of COVID-19 ranges from asymptomatic to dry cough, fever, dyspnea, headache, life-threatening pneumonia, or ARDS resulting in respiratory failure and death [2,3]. The elderly and patients with other medical comorbidities are more prone to severe respiratory disease [4]. The varied clinical presentation and considering susceptible individuals have a short duration for the onset of ARDS with high mortality rates, and therefore early diagnosis and management are vital [2,4]. Blood investigations contribute to early diagnosis by providing information about the inflammatory process [4]. The complete blood count (CBC) is a low-cost and easy to perform test [4]. It provides information regarding total leukocyte count (TLC), differential leukocyte count (DLC), platelet count, and indices [4]. Some calculated ratios from these parameters may act as inflammatory markers [4]. Previous studies have shown that CBC parameters have clinical importance in predicting disease outcomes. Most of these studies are based on the Chinese population. However, there is a paucity of published data from our region in India. The present study described the epidemiological, clinical, and hematological characteristics and outcomes of COVID-19 confirmed cases.

## Materials and methods

This study was a descriptive study approved by the Institutional Ethical Committee. The study aimed to know the epidemiological, clinical, and hematological characteristics of COVID-19 confirmed cases. We also tried to find the association of hematological parameters with the outcomes of these cases. All real-time reverse transcriptase-polymerase chain reaction (RT-PCR) confirmed SARS-CoV-2 cases on nasopharyngeal swabs admitted in our institute over a period of three months, from July to September 2020, were included in the study population. Patients with a history of blood transfusion within the last month, those receiving blood transfusions during hospitalization, or those with any hematological diseases were excluded from the study. The blood samples of SARS-CoV-2 positive cases were analyzed on admission and at the time of discharge (most recent in case of mortality). The patients' hematological parameters were evaluated using a fully automated Sysmexä XN-1000 six-part differential analyzer. Hemoglobin, TLC, DLC, absolute neutrophil count (ANC), absolute lymphocyte count (ALC), absolute eosinophil count (AEC), absolute monocyte count (AMC), neutrophil-to-lymphocyte ratio (NLR), platelet-to-lymphocyte ratio (PLR), and platelet count were recorded. Activated partial thromboplastin time (APTT) and prothrombin time (PT) were obtained by electromechanical clot detection method, while D-dimer was measured by immuno-turbidimetric assay on Stago STA Compact Max®. The epidemiological data, relevant patient history, clinical details, severity (intensive care unit [ICU]/non-ICU), and patient outcome (recovery/death) were recorded from patient datasheets.

Data analysis

The software SPSS Version 22 (IBM Corp., Armonk, NY) was used for statistical analysis of the data. Statistical analysis considering the nature of variables was performed for descriptive statistics. Proportion and frequency were used for data presentation. The chi-square test was used for finding out the association between the hematological parameters of ICU versus non-ICU groups. A p-value of less than 0.05 was considered significant at 95% CI and appropriate degree of freedom.

## Results

A total of 252 RT-PCR confirmed SARS-CoV-2 cases were included in the study. The most common age group affected was 46 to 60 years (94/252) followed by 61 to 75 years (73/252). Males were affected more compared to females, with a male-to-female ratio of 2.45:1 (179/73). The rural-to-urban ratio was 1.05:1 (129/123). History of travel and contact history were present in 98.81% (249/252) and 93.65% (236/252), respectively. The most common clinical symptom was dyspnea (60.71% [153/252] followed by fever (46.03% [116/252]). The commonest comorbidity was hypertension (38.89% [98/252]) followed by diabetes (34.13% [86/252]) (Table 1).

**Table 1:** Clinical and epidemiologic parameters in COVID-19 positive ICU versus non-ICU and death versus recovered cases. COVID-19, coronavirus disease 2019; ICU, intensive care unit

Variable	Category	ICU	Non-ICU	Test of significance (p-value)	Death	Recovered	Test of significance (p-value)
Age	1-15 years	3 (37.5%)	5 (62.5%)	0.003	1 (12.5%)	7 (87.5%)	0.016
	16-30 years	1 (5.3%)	18 (94.7%)		0 (0%)	19 (100%)	
	31- 45 years	15 (33.3%)	30 (66.7%)		5 (11.1%)	40 (88.9%)	
	46- 60 years	32 (34.0%)	62 (66.0%)		15 (16.0%)	79 (84.0%)	
	61- 75 years	39 (53.4%)	34 (46.6%)		20 (26.4%)	53 (73.6%)	
	>76 years	7 (53.8%)	6 (46.2%)		5 (38.5%)	8 (61.5%)	
Gender	Male	81 (45.3%)	98 (54.7%)	0.001	37(20.7%)	142 (79.3%)	0.120
	Female	16 (21.9%)	57 (78.1%)		9 (12.3%)	64 (87.7%)	
Residence	Urban	50 (40.7%)	73 (59.3%)	0.492	23(18.7%)	100 (81.3%)	0.858
	Rural	47 (36.4%)	82 (63.6%)		23 (17.8%)	106 (82.2%)	
Travel history	Yes	96 (38.6%)	153 (61.4%)	0.853	1(33.3%)	2 (66.7%)	0.496
	No	1 (33.3%)	2 (66.7%)		45 (18.1%)	204 (81.9%)	
Contact history	Yes	92(39.0%)	144 (61.0%)	0.538	3(18.8%)	13 (81.2%)	0.958
	No	5 (31.2%)	11 (68.8%)		43 (18.2%)	193 (81.8%)	
Fever	Yes	73 (62.93%)	43 (37.07%)	0.600	35 (30.17%)	81 (69.83%)	0.650
	No	24 (35.5%)	112 (60.5%)		11 (8.1%)	125 (91.9%)	
Headache	Yes	4 (50.0%)	4 (50.0%)	0.497	2 (25.0%)	6 (75.0%)	0.497
	No	93 (38.1%)	151 (61.9%)		44 (18.0%)	200 (82.0%)	
Dyspnea	Yes	80 (52.3%)	73 (47.7%)	<0.001	34 (22.2%)	119 (77.8%)	0.616
	No	17 (17.2%)	82 (82.8%)		12 (12.1%)	87 (87.9%)	
Loss of taste	Yes	2 (40.0%)	3 (60.0%)	0.930	0 (0%)	5 (100.0%)	0.566
	No	95 (38.5%)	152 (61.5%)		46 (18.6%)	201 (81.4%)	
Diabetes	Yes	39 (45.3%)	47 (54.7%)	0.107	21 (24.4%)	65 (75.6%)	0.068
	No	58 (34.9%)	108 (65.1%)		25 (15.1%)	141 (84.9%)	
Hypertension	Yes	43 (43.9%)	55 (56.1%)	0.161	21 (21.4%)	77 (78.6%)	0.298
	No	54 (35.1%)	100 (64.9%)		25 (16.2%)	129 (83.8%)	
Cardiovascular disease	Yes	8 (40.0%)	12 (60.0%)	0.885	3 (15.0%)	17 (85.0%)	0.695
	No	89 (38.4%)	143 (61.6%)		43 (18.5%)	189 (81.6%)	
Pulmonary disease	Yes	3 (20.0%)	12 (80.0%)	0.129	2 (13.3%)	13 (86.7%)	0.611
	No	94 (39.7%)	143 (60.3%)		44 (18.6%)	193 (81.4%)	
Liver disease	Yes	6 (66.7%)	3 (33.3%)	0.077	4 (44.4%)	5 (55.6%)	0.038
	No	91 (37.4%)	152 (62.6%)		42 (17.3%)	201 (82.7%)	
Chronic renal disease	Yes	7 (41.18%)	10 (58.82%)	0.814	6 (35.3%)	11 (64.7%)	0.060
	No	90 (38.3%)	145 (61.7%)		40 (20.5%)	195 (79.5%)	
Malignancy	Yes	2 (33.3%)	4 (66.7%)	0.793	2 (33.3%)	4 (66.7%)	0.333
	No	95 (38.6%)	151 (61.4%)		44 (17.9%)	202 (82.1%)	

The D-dimer results were available in 226 cases. The ICU admission was higher in patients associated with neutrophilia, lymphopenia, monocytopenia, thrombocytopenia, and elevated D-dimer levels. While recovery rate was also greater in these cases except, increased D-dimer was related to increased mortality (Table 2).

**Table 2:** Association of hematological parameters in COVID-19 positive ICU versus non-ICU and death versus recovered cases. AEC, absolute eosinophil count; ALC, absolute lymphocyte count; AMC, absolute monocyte count; ANC, absolute neutrophil count; COVID-19, coronavirus disease 2019; ICU, intensive care unit; NLR, neutrophil-to-lymphocyte ratio; PLR, platelet-to-lymphocyte ratio

Variable	Category	ICU	Non-ICU	Test of significance (p-value)	Death	Recovered	Test of significance (p-value
ANC	Normal	30 (24.4%)	93 (75.6%)	<0.001	10 (8.1%)	113 (91.9%)	<0.001
	Neutropenia	0 (0%)	5 (100%)		1 (20.0%)	4 (80.0%)	
	Neutrophilia	67 (54%)	57 (46%)		35 (28.2%)	89 (71.8%)	
ALC	Normal	46 (30.7%)	104 (69.3%)	0.071	18 (12.0%)	132 (88%)	0.004
	Lymphocytosis	2 (33.3%)	4 (66.7%)		0 (0%)	6 (100%)	
	Lymphopenia	42 (53.2%)	37 (46.8%)		24 (30.4%)	55 (69.6%)	
	Severe lymphopenia	7 (41.2%)	10 (58.8%)		4 (23.5%)	13 (76.5%)	
AEC	Normal	87 (43.5%)	114 (56.5%)	0.007	42 (20.9%)	159 (79.1%)	0.031
	Eosinophilia	10 (19.6%)	41 (80.4%)		4 (7.8%)	47 (92.2%)	
AMC	Normal	32 (40.5%)	47 (59.5%)	0.469	15 (19.0%)	64 (81.0%)	0.705
	Monocytosis	2 (66.7%)	1 (33.3%)		0 (0%)	3 (100.0%)	
	Monocytopenia	63 (37.1%)	107 (62.9%)		31 (18.2%)	139 (81.8%)	
Platelet count	Normal	128 (62.2%)	78 (37.8%)	0.938	38 (18.4%)	168 (81.6%)	0.780
	Thrombocytosis	5 (50%)	5 (50%)		1 (10%)	9 (90%)	
	Thrombocytopenia	22 (61.1%)	14 (38.9%)		7 (19.4%)	29 (80.6%)	
D-dimer (226 cases)	Normal	13 (21.7%)	47 (78.3%)	<0.001	54 (90.0%)	6 (10.0%)	0.046
	Abnormal	75 (45.2%)	91 (54.8%)		130 (78.3%)	36 (21.7%)	
Variable	Cut-off value	ICU	Non-ICU	Test of significance (p-value)	Death	Recovered	Test of significance (p-value)
NLR	<3.17	10 (14.7%)	58 (85.3%)	<0.001	4 (5.9%)	64 (94.1%)	0.002
	>3.17	87 (47.3%)	97 (52.7%)		42 (22.8%)	142 (77.2%)	
PLR	<150	41 (28.5%)	103 (71.5%)	0.002	20 (13.9%)	124 (86.1%)	0.038
	>150	56 (51.9%)	52 (48.1%)		26 (24.1%)	82 (75.9%)	
Variable	Anemia	ICU	Non-ICU	Test of significance (p-value)	Death	Recovered	Test of significance (p-value)
Male	Anemic	43 (37.7%)	71 (62.3%)	0.824	22 (19.3%)	92 (80.7%)	0.332
	Normal	26 (39.4%)	40 (60.6%)		9 (13.6%)	57 (86.4%)	
Female	Anemic	19 (33.9%)	37 (66.1%)	0.106	10 (17.9%)	46 (82.1%)	0.245
	Normal	9 (56.2%)	7 (43.8%)		5 (31.2%)	11 (68.8%)	

The statistical analysis of the association between ICU versus non-ICU patients and mortality (death) versus recovered cases was performed with clinical, epidemiological, and hematological parameters. The analysis showed a significant association of age, ANC, AEC, D-dimer, NLR, and PLR with ICU versus non-ICU patients and death versus recovered cases. Gender and dyspnea showed significant association with ICU versus non-ICU patients only, while comorbidity of liver disease and ALC had a significant association with death in comparison to recovered cases. NLR was also significant in males compared to females in COVID-19 positive cases.

## Discussion

SARS-CoV-2 causing COVID-19 has infected millions of people in India and worldwide, affecting all age groups [1,2]. The median age varies from 40 to 68 years, with male predominance in some research [1,2,4-6]. In symptomatic patients, the common clinical symptoms were fever, sore throat, cough, myalgia, fatigue, dyspnea, headache, shortness of breath, chest pain, abdominal pain, diarrhea, nausea, vomiting, and rhinorrhea [2,4]. The common comorbidity encountered in these patients were chronic obstructive pulmonary disease (COPD), hypertension, diabetes mellitus, cardiovascular disease, and malignancy [2,6,7]. Age, hypertension, diabetes mellitus, cardiovascular disease, and COPD show the significant clinical difference and are positively correlated with in-hospital mortality [6,8,9]. Compared to patients who did not require ICU care, those who required ICU care were also significantly associated with age, hypertension, diabetes mellitus, cardiovascular disease, malignancy, cerebrovascular disease, and COPD [7]. Older age and the presence of comorbidity are common among severe disease cases [10]. ICU care is also associated with older age, and ICU patients were about a decade older than the non-ICU patients [7,11]. However, Huang et al. did not find the association of age and comorbidities with those who required ICU care [12]. In the present study, we also observed male predominance, with the highest incidence of COVID-19 infection in the 46- to 60-year age groups, and 61- to 75-year age group was the second most common group. Dyspnea and fever were the common clinical symptoms, while hypertension and diabetes were common comorbidities. We also observed a significant association between age with ICU admission and death in COVID-19 cases. At the same time, there was a significant relationship between gender and ICU care, with 45.3% males requiring ICU admission. Dyspnea (52%) was the symptom significantly associated with ICU admission, and among the underlying comorbidity, liver disease was most associated with death.

Similar to Sun et al., we also speculate that in COVID-19 positive patients, leukopenia occurred due to the migration of leukocytes from peripheral blood to lung tissue and other organs of injury [13]. Leukopenia in COVID-19 patients has also been reported in various reports [14,15]. Neutrophils are seen in various lung disorders, including respiratory viral infections, leaving the systemic circulation, and getting activated to cause damage to pulmonary vasculature and interstitium [13,16,17]. In COVID-19 cases, neutrophilia is probably a chemotaxis response to the cytokine storm and positively correlates with death [3,6,8]. In COVID-19 cases, the neutrophilia is varied from 4% to 38.8% [1,3,18,19]. In our study, increased neutrophil counts were found in 114 (45.2%) cases and were statistically significant with the severity of the infection.

SARS-CoV-2 may cause apoptosis or necrosis of lymphocytes resulting in lymphopenia [1]. Lymphopenia, a common finding in viral infection, is also commonly reported in COVID-19 patients [1-6]. Lymphopenia was observed in previous studies and occurred in 25% to 83.2% of cases [1,10,20]. The percentage of lymphocyte and neutrophil is significantly correlated with SARS-CoV-2 viral load, and the severity of COVID-19 is consistently correlated with lymphopenia [1,21]. Fan et al. observed that COVID-19 patients that required ICU care had significantly lower ALC [11]. Wang et al. and Zhou et al. also observed that lymphopenia is more common in-hospital death than recovered COVID-19 cases [7,9]. Zhang et al. observed that higher neutrophil and lower lymphocyte percentages were strongly related to severe COVID-19 pneumonia and composite endpoints (ICU admission, mechanical ventilation, or death) [22]. Lymphocyte may be a biomarker to predict the outcome and death in COVID-19 patients [1,8]. NLR may also act as an independent risk factor, especially for males, and may also help identify high-risk individuals COVID-19 [1,8]. NLR is a cost-effective, easily measurable, reliable parameter and proposed as a new biomarker for systemic inflammatory response. Continuous NLR monitoring may be helpful in the diagnosis and management of COVID-19 cases [6,8]. Like previous studies, we also observed a significant association between neutrophilia and death and those who needed ICU care. In our research, lymphopenia showed a significant association between in-hospital death and recovered COVID-19 patients. NLR also showed a significant association with death and those who required ICU admission.

Thrombocytopenia is one of the common hematological findings observed in the COVID-19 cases [23]. The increased platelet clearance, along with increased platelet consumption, lung damage, dysfunctional bone marrow microenvironment, decreased thrombopoietin production, and antiviral drug use is the possible vital factor for thrombocytopenia COVID-19 cases [23]. The direct attack on hematopoietic stem/progenitor cells by SARS-CoV-2, injury to the lung, and increased platelet destruction by autoantibodies/immune complexes formed in response to SARS-CoV-2 may also cause thrombocytopenia [23]. Thrombocytopenia is associated with a high risk of severe COVID-19 and an increased risk of in-patient death [9,10,23-26]. The platelet count monitoring may act as a clinical pointer for the worsening and prognosis of the disease [25,26]. The low platelet count was reported in 5% to 53.6% of COVID-19 cases [1,12,20]. Seyit et al. observed significantly higher levels of NLR and PLR in COVID-19 cases [27]. Yang et al. studied the 93 COVID-19 cases, observed high PLR in severe cases, and considered it an independent factor associated with COVID-19 progression [28]. However, after adjustment of gender and age, the statistical significance of PLR is not clear [28]. Higher PLR value is associated with the prognosis of COVID-19 and increased duration of hospital stay [29]. During treatment and PLR at platelet peak, the platelet peak shows a significant difference in severe and non-severe patients. PLR at platelet peak during management might be an independent influencing factor for severe cases [29]. The present study observed that thrombocytopenia was more in those who required ICU care. However, there was no significant statistical association between severity of infection or recovery versus mortality. However, lower PLR showed a significant association with non-ICU care and higher recovery.

The role of monocytes and macrophages in the pathophysiology of COVID-19 is not clear, but they may act as a vital component of the inflammatory response to SARS-CoV-2 [30]. Stimulation with the S protein polarizes the THP-1 macrophages toward proinflammatory states, with an increase in the TNFα and MHC-II expression, which may be used as potential therapeutic targets [31]. The severe cases of COVID-19 have lower percentages of monocytes, and AMC is inversely associated with increased mortality [5,32]. Liu et al. studied 245 cases of COVID-19 and noted the significant association of baseline monocyte count with mortality in the unadjusted model, which was lost after adjusting cofounders [8]. The statistically significant decrements in AMC are also reported in the ICU group as compared with the non-ICU group [11]. However, Wang et al. found no association of monocyte count in between ICU versus non-ICU cases [7]. In the present study, monocytopenia was observed in 25% of cases, but there was no AMC association when we compared ICU versus non-ICU and death versus recovered patients.

Eosinopenia is reported in the early phase of SARS-CoV-2 infection and severe cases [3,13,32,33]. Seyit et al. studied 233 cases of suspected COVID-19 cases and observed a significant increase of eosinophils in COVID-19 negative cases compared with positive cases [27]. Fraissé et al. analyzed 78 COVID-19 cases and observed that 69 patients had eosinopenia at ICU admission [33]. Twenty-six cases that developed eosinophilia during ICU stay include 22 cases that had eosinopenia at ICU admission [33]. They also observed the association of eosinophilia with a lower death rate in ICU [33]. Eosinophilia may be the direct or indirect response of SARS-CoV-2 because of infection or recovery from it [33]. Tan et al. studied 40 cases of COVID-19 and observed that 82.5% of cases were reported with low eosinophils count at the time of admission [34]. Using Spearman's correlation coefficient, they found an inverse relation of lymphocytes and eosinophils with the severity of the disease [34]. They also stated that for the diagnosis of COVID‐19, eosinophils have better sensitivity and play a significant role in assessing the prognosis of patients like lymphocytes [34]. The increase in eosinophils count on day 7 of hospitalization is associated with a better prognosis and lower complication rates [35]. In this study, eosinophilia was significantly increased in non-ICU and recovered cases.

Some studies support the presence of a syndrome of COVID-19 associated coagulopathy characterized by abnormal PT, APTT, higher D-dimer levels, and an increased thrombotic propensity [3]. The literature reveals significantly elevated PT and D-dimer in severe COVID-19 cases [36]. They were also associated with poor prognosis when elevated at the time of admission and commonly observed in patients who require ICU care [7,12]. Saurabh et al. observed that D-dimer ≥ 0.99 μg/mL predicted an increase in disease severity, with a sensitivity of 97% and specificity of 86% [37]. D-dimer is significantly elevated in patients who died when compared with survivors [38]. Zhang et al. used the D-dimer cut-off value of ≥2 μg/mL and observed a specificity of 83.3% and sensitivity of 92.3% to predict in-patient mortality [39]. Zhou et al. observed that a higher D-dimer level at admission was one of the risk factors for mortality in COVID-19 adult cases [9]. At an early stage of COVID-19 infection, D-dimer >1 μg/mL may help clinicians discriminate against patients with poor prognosis [12]. We studied the D-dimer in 226 cases and found its significant association in both non-survival versus survival (p = 0.046) and ICU versus non-ICU cases (p = 0.001).

An adequate hemoglobin level is required to ensure sufficient tissue oxygenation [1]. Those patients who become severe during their stay have a significant decrease in their hemoglobin levels compared to the non-ICU patient [11]. Zhou et al. found a significant difference in anemia between non-survival and survival COVID-19 patients [9]. However, we did not observe any significant association of anemia between ICU versus non-ICU and non-survival and survival COVID-19 cases.

Limitation

The main limitation of this study was the smaller number of patients. Studies conducted with a larger patient group will better elaborate the importance of peripheral blood tests in the diagnosis and prognosis of COVID-19 patients. Another limitation was not including the personal habits (e.g., cigarette smoking and alcohol use), and their effects on the results were not accounted for.

## Conclusions

The COVID-19 disease has had many notable clinical and hematological manifestations. Age, gender, dyspnea, liver disease, ANC, ALC AEC, NLR, PLR, and D-dimers were the common clinical and hematological parameters that may predict the progression and outcome of the COVID-19 disease. Continuous and regular monitoring of these parameters is recommended, which may help the clinician predict disease progression, reduce the risk of mortality, and improve the quality of life.
